# Detection of the *iroN* virulence gene in multidrug-resistant *Escherichia coli* isolated from quails in traditional markets of Surabaya, Indonesia

**DOI:** 10.14202/vetworld.2026.920-932

**Published:** 2026-03-12

**Authors:** Maria Oliva Keytimu, Ummi Rahayu, Freshinta Jellia Wibisono, Mustofa Helmi Effendi, John Yew Huat Tang, Mariana Febrilianti Resilinda Putri, Aswin Rafif Khairullah, Saifur Rehman, Wasito Wasito, Riza Zainuddin Ahmad, Bima Putra Pratama, Irfan Alias Kendek

**Affiliations:** 1 Master Program of Veterinary Science and Public Health, Faculty of Veterinary Medicine, Universitas Airlangga, Jl. Dr. Ir. H. Soekarno,Kampus C Mulyorejo, Surabaya, 60115, East Java, Indonesia; 2 Department of Veterinary Public Health, Faculty of Veterinary Medicine, Universitas Wijaya Kusuma Surabaya, Jl. Dukuh Kupang XXV No.54, Dukuh Kupang, Dukuh Pakis, Surabaya, 60225, East Java, Indonesia; 3 Department of Veterinary Public Health, Faculty of Veterinary Medicine, Universitas Airlangga, Jl. Dr. Ir. H. Soekarno, Kampus C Mulyorejo, Surabaya, 60115, East Java, Indonesia; 4 Research Group of Antimicrobial Resistance in Faculty of Veterinary Medicine, Universitas Airlangga Surabaya, East Java, Indonesia; 5 School of Food Industry, Faculty of Bioresources, and Food Industry, Universiti Sultan Zainal Abidin (Besut Campus), Besut, 22200, Malaysia; 6 Department of Anatomy, Physiology, Pharmacology, Biochemistry (AFFB), Faculty of Medicine and Veterinary Medicine, Universitas Nusa Cendana, Jl. Adisucipto Penfui, Kupang, 85221, East Nusa Tenggara, Indonesia; 7 Research Center for Veterinary Science, National Research and Innovation Agency (BRIN), Jl. Raya Bogor Km. 46 Cibinong, Bogor, 16911, West Java, Indonesia; 8 Department of Pathobiology, Faculty of Veterinary and Animal Sciences, Gomal University, RV9W+GVJ, Indus HWY, Dera Ismail Khan, 27000, Pakistan; 9 Research Center for Process Technology, National Research and Innovation Agency (BRIN), Jl. Raya Puspiptek 60, South Tangerang, 15310, Banten, Indonesia; 10 Department of Microbiology, Faculty of Health, Pharmacy Study Program, Universitas Sari Mulia, Jl. Pramuka No. 2, Banjarmasin, 70238, South Kalimantan, Indonesia

**Keywords:** antimicrobial resistance, avian pathogenic *Escherichia coli*, *E. coli*, traditional markets, *iroN* virulence gene, polymerase chain reaction, public health

## Abstract

**Background and Aim:**

*Escherichia coli* is a common intestinal commensal in poultry, but avian pathogenic *E. coli* (APEC) strains can cause colibacillosis and pose zoonotic risks due to genetic similarities with human extraintestinal pathogenic *E. coli* (ExPEC). Quails sold in traditional markets may serve as reservoirs for multidrug-resistant (MDR) and virulent strains, yet data from Indonesia are limited. Iron acquisition systems, such as the *iroN* gene encoding the salmochelin siderophore receptor, are critical virulence determinants in APEC, enabling survival in iron-limited host environments and potentially linking to antimicrobial resistance (AMR). This cross-sectional laboratory-based study aimed to detect MDR *E. coli* from quail cloacal swabs in Surabaya’s traditional markets and screen MDR isolates for the *iroN* gene, highlighting market level risks within a One Health framework.

**Materials and Methods:**

From November to December 2024, 150 cloacal swabs were collected from quails across five traditional markets (Turi, Bratang, Cemara Pabean, Kupang, and Benowo) in Surabaya, Indonesia. Samples were enriched in buffered peptone water, streaked on eosin methylene blue agar and MacConkey agar, and confirmed as *E. coli* via Gram staining and biochemical tests (Triple Sugar Iron Agar, Simmons Citrate Agar, Sulfide Indole Motility, and Methyl Red–Voges Proskauer). Antibiotic susceptibility was assessed using the Kirby–Bauer disk diffusion method on Mueller–Hinton agar against aztreonam (ATM 30 µg), ciprofloxacin (CIP, 5 µg), tetracycline (TE, 30 µg), kanamycin (K, 30 µg), and chloramphenicol (C, 30 µg), interpreted per Clinical and Laboratory Standards Institute M100 (2023) guidelines. MDR was defined as resistance to ≥3 antibiotic classes. MDR isolates underwent polymerase chain reaction for *iroN* detection.

**Results:**

*E. coli* was isolated from 148/150 samples (98.7%), with 100% positivity in Turi, Bratang, and Cemara Pabean markets. Resistance rates were highest to C (33.1%), followed by TE (22.3%), ATM (13.5%), K (6.1%), and C (4.7%). Four isolates (2.7%) were MDR, distributed in Turi (1), Cemara Pabean (2), and Kupang (1). MDR patterns included ATM/CIP/TE (two isolates), ATM/CIP/K (one), and ATM/CIP/TE/K/C (one). All four MDR isolates were positive for *iroN*, indicating a 100% association in this subset.

**Conclusion:**

Quails in Surabaya’s traditional markets harbor prevalent *E. coli* with notable AMR, including MDR strains carrying the *iroN* virulence gene, underscoring their role as potential APEC reservoirs. This convergence of resistance and virulence highlights zoonotic and public health risks, necessitating enhanced AMR surveillance, market hygiene, and antibiotic stewardship under One Health principles. Future studies should explore genomic mechanisms and transmission pathways.

## INTRODUCTION

*Escherichia coli* is a Gram-negative bacterium that naturally inhabits the digestive tract of warm-blooded animals, including poultry [1]. Although most strains are commensal, some variants possess virulence factors that enable serious infections to occur outside the digestive tract [2]. Avian pathogenic *Escherichia coli* (APEC) is one of the primary agents of colibacillosis in poultry, leading to substantial economic losses through reduced production performance, increased antibiotic use, and mortality [3]. Beyond its impact on animal health, APEC holds zoonotic relevance due to its genetic similarity to extraintestinal pathogenic *E. coli* (ExPEC) in humans, positioning poultry as a potential reservoir for pathogenic strains that can cross-species barriers [4].

Quails differ from broilers and layers in several key aspects relevant to antimicrobial resistance (AMR) and pathogen dissemination. As short-cycle poultry, they are often reared on smallholder farms with variable antimicrobial usage practices [5]. Their distribution and sales predominantly occur through traditional markets, such as those in Surabaya. Unlike farm-level systems, traditional markets serve as the final node in the poultry value chain, where animals from diverse sources are aggregated, heightening the risks of bacterial mixing, cross-contamination, and human exposure. These markets typically feature high animal densities, limited sanitation, and rapid turnover, fostering an environment conducive to the spread of enteric bacteria [6]. Consumers, vendors, and the surrounding environment may face direct exposure to pathogenic and antimicrobial-resistant bacteria via handling of live birds, contaminated surfaces, aerosols, and improper waste disposal, underscoring the importance of traditional markets as critical surveillance points within a One Health framework [7].

APEC pathogenicity is mediated by a diverse array of virulence factors, including adhesins, serum resistance proteins (e.g., iss), outer membrane proteins (ompA), toxins (hlyF), and iron acquisition systems [11, 12]. This study prioritizes iron acquisition genes because they are essential for bacterial survival in iron-limited host environments and are strongly linked to systemic infections, environmental persistence, and enhanced fitness under stress conditions. Iron is tightly regulated in the host via iron-binding proteins such as transferrin and lactoferrin, establishing a nutritional immunity barrier against bacterial proliferation [13]. To circumvent this, APEC has evolved multiple iron uptake mechanisms, encompassing siderophore-mediated systems (enterobactin, aerobactin, and salmochelin), ferrous iron transporters, and heme utilization pathways [14]. Genes involved in these systems, particularly *iroN*, which encodes the salmochelin siderophore receptor, have been frequently associated with highly virulent and zoonotically relevant APEC and ExPEC strains, rendering them valuable molecular markers for risk assessment [15]. Iron acquisition genes may co-occur with AMR determinants, potentially augmenting bacterial adaptability and persistence under antimicrobial pressure [16–18].

However, studies on the characteristics of *E. coli* from quails, particularly in the context of traditional markets in Indonesia, remain limited. Most existing surveillance efforts focus on broiler or layer chickens and are predominantly conducted at the farm-level, leading to a significant dearth of data on quail-derived *E. coli* circulating at market interfaces [4]. As a result, the prevalence of multidrug-resistant (MDR) *E. coli* and its associated virulence determinants in quails sold in traditional Indonesian markets is largely unknown. This knowledge deficit is exacerbated by the ongoing development of AMR [8], driven by antibiotic use in the livestock sector for both therapeutic and growth-promoting purposes, which selectively favors resistant bacteria [9]. MDR *E. coli* strains, defined as those resistant to three or more antibiotic classes—represent a profound health threat due to their capacity for dissemination and diminished therapeutic efficacy [10]. Furthermore, while iron acquisition genes like *iroN* are recognized for their role in virulence, no study to date has explicitly investigated the association between MDR phenotypes and these genes in *E. coli* isolated from quails in traditional Indonesian markets. This oversight highlights a critical gap in understanding how AMR and virulence traits converge at market level interfaces, potentially amplifying zoonotic risks and undermining One Health strategies.

Based on the available evidence, we hypothesized that MDR *E. coli* isolates from quails in traditional markets are more likely to harbor iron-related virulence genes, particularly *iroN*. This study uniquely characterizes MDR *E. coli* and iron acquisition virulence traits at the traditional market interface, which represents a pivotal yet understudied node in the One Health transmission continuum. Therefore, this study aimed to detect iron acquisition genes in MDR *E. coli* isolated from cloacal swabs of quails collected at traditional markets in Surabaya. By emphasizing market level surveillance, this research provides insights into real-world exposure pathways for humans and the environment, thereby supporting evidence-based strategies for AMR risk mitigation within a One Health framework.

## MATERIALS AND METHODS

### Ethical approval

Ethical approval for this study was obtained from the Animal Ethics Committee, Faculty of Veterinary Medicine, Universitas Wijaya Kusuma Surabaya, Indonesia (Ethical Approval No.: 170-KKE-2025). Permission and informed consent were obtained from quail owners or vendors before cloacal swab collection. To minimize animal stress and discomfort, all sampling procedures were performed by trained personnel using gentle manual restraint, and no invasive procedures or sedation were applied during sample collection.

### Study period and location

This cross-sectional, laboratory-based observational study was conducted from November to December 2024 to assess MDR *E. coli* and iron-related virulence genes in quails at the market level.

### Research design

A total of 150 cloacal swab samples were collected, with the sample size determined based on feasibility considerations and previous poultry AMR surveillance studies, which typically use 100–200 samples to estimate prevalence at the market level. This sample size was deemed sufficient to provide an initial estimation of the occurrence of MDR *E. coli* in quails sold in traditional markets in Surabaya.

Samples were obtained from quails sold in five traditional markets (Turi, Bratang, Cemara Pabean, Kupang, and Benowo) in Surabaya, Indonesia. These markets were purposively selected as major traditional poultry trading centers, geographically distributed across Surabaya, and serving as primary supply points for live quails to the local population, making them representative of market level quail distribution within the city.

A random sampling procedure was applied at the vendor level within each market. On the sampling day, vendors selling live quails were enumerated, and to minimize selection bias, individual quails were selected using a simple random technique (every nth bird). Only apparently healthy quails intended for sale, regardless of sex, were included, while visibly sick, injured, or dead birds were excluded to ensure sample quality. Information on age and farm-origin was not available at the market level and thus could not be used as an inclusion criterion.

The swabs were placed in sterile transport media and transported in a cooled container to the Veterinary Public Health Laboratory, Faculty of Veterinary Medicine, Universitas Wijaya Kusuma Surabaya, for further analysis.

### Isolation and identification of *E. coli*

Cloacal swab samples were first enriched in buffered peptone water and incubated aerobically at 37°C for 18–24 h. The enriched samples were then streaked onto eosin methylene blue agar (EMBA) and MacConkey agar plates and incubated aerobically at 37°C for 24 h. Colonies exhibiting a characteristic green metallic sheen on EMBA or lactose-fermenting pink colonies on MacConkey agar were presumptively identified as *E. coli*.

Presumptive colonies were further examined by Gram staining and confirmed using a series of biochemical tests, including Triple Sugar Iron Agar (TSIA), Simmons Citrate Agar (SCA), Sulfide Indole Motility (SIM), and Methyl Red–Voges Proskauer (MR–VP), following standard protocols [19, 20]. Reference quality control strains, including *E. coli* ATCC 25922, were used in parallel to ensure the accuracy of culture and biochemical tests. All ambiguous or atypical colonies were retested in duplicate to confirm their identity and ensure repeatability.

### Antibiotic resistance testing

Antibiotic susceptibility testing was performed using the Kirby–Bauer disk diffusion method, in which bacterial suspensions were adjusted to a 0.5 McFarland turbidity standard and uniformly spread onto Mueller–Hinton agar plates. Antibiotic-impregnated disks were aseptically placed on the agar surface, followed by aerobic incubation at 37°C for 18–24 h. The tested antibiotics were selected based on their clinical relevance in poultry and human medicine and their common usage in the local poultry sector.

The following antibiotics and their disk concentrations were used: aztreonam (ATM, 30 µg), ciprofloxacin (CIP, 5 µg), tetracycline (TE, 30 µg), kanamycin (K, 30 µg), and chloramphenicol (C, 30 µg). Inhibition zone diameters were measured in millimeters and interpreted according to the specific breakpoints provided in the Clinical and Laboratory Standards Institute (CLSI) document M100, 2023 edition [21], for Enterobacteriaceae. Quality control was performed using *E. coli* ATCC 25922 in parallel with the test isolates.

Bacterial isolates were classified as resistant, intermediate, or susceptible based on CLSI standards. MDR was defined as resistance to three or more antibiotic classes, grouped as follows: β-lactams (ATM), fluoroquinolones (CIP), tetracyclines (TE), aminoglycosides (K), and phenicols (C). This definition reflects phenotypic MDR, indicating the isolate’s resistance profile, and does not necessarily correspond to clinical treatment failure but provides a standardized measure to identify high-risk isolates for surveillance purposes [22, 23].

### Polymerase chain reaction (PCR)

PCR was used to detect the iron acquisition gene *iroN* in *E. coli* isolates. Genomic DNA was extracted from overnight pure bacterial colonies grown on nutrient agar using a commercial bacterial DNA extraction kit according to the manufacturer’s instructions. DNA concentration and purity were measured using a spectrophotometer prior to PCR amplification. PCR reactions were prepared in a total volume of 25 µL, consisting of 12.5 µL of 2× PCR master mix (including Taq DNA polymerase, 1.5 mM MgCl_2_ and 200 µM dNTPs), 0.5 µM of each primer, 2 µL of template DNA (~50 ng), and nuclease-free water to final volume.

The primer set for *iroN* was as follows: forward: GAGTATTCAACATTTCCGTGTC, reverse: TAATCAGTGAGGCA CCTATCTC, targeting an 861 bp fragment with an annealing temperature of 57°C [24]. The PCR cycling conditions were initial denaturation at 94°C for 5 min, followed by 35 cycles of denaturation at 94°C for 30 s, annealing at 57°C for 30 s, extension at 72°C for 1 min, and final extension at 72°C for 10 min. Amplicons were visualized by electrophoresis on a 1.5% agarose gel stained with ethidium bromide and viewed under UV light. Positive and negative controls were included in each run to validate the results.

## RESULTS

### Isolation and identification of *E. coli*

**Figure 1:**
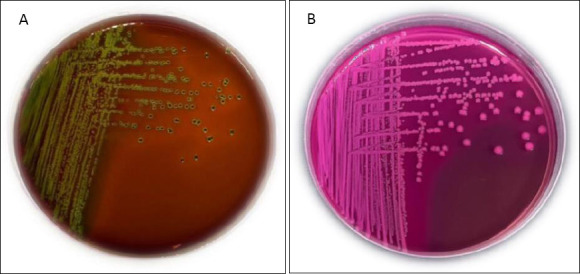
(A) *Escherichia coli* colonies on eosin methylene blue agar exhibiting a characteristic metallic green sheen. (B) *E. coli* colonies on MacConkey agar appearing as pink to red lactose-fermenting colonies.

**Figure 2:**
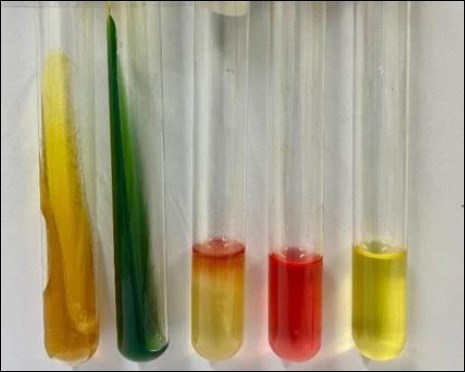
Biochemical testing of *Escherichia coli* bacterial isolates: (1) triple sugar iron agar, (2) Simmons citrate agar, (3) sulfide indole motility, (4) methyl red, and (5) Voges Proskauer.

*Escherichia coli* was isolated from 148 out of 150 cloacal swab samples, yielding an overall prevalence of 98.7%. Isolation rates varied slightly across the sampled traditional markets: 100% (30/30) in Turi, Bratang, and Cemara Pabean; 96.7% (29/30) in Kupang; and 96.7% (29/30) in Benowo (Table 1). Characteristic colonies on EMBA displayed a green metallic sheen, while those on MacConkey agar appeared as pink lactose-fermenting colonies (Figures 1A and 1B). Biochemical confirmation via TSIA, SCA, SIM, and MR–VP tests further validated the isolates as *E. coli* (Figure 2).

**Table 1:** *Escherichia coli* isolation and identification.

Traditional market	Number of samples collected	*E. coli* (%) positive	Negative
Turi	30	100% (30/30)	0% (0/30)
Bratang	30	100% (30/30)	0% (0/30)
Cemara Pabean	30	100% (30/30)	0% (0/30)
Kupang	30	96.7% (29/30)	3.3% (1/30)
Benowo	30	96.7% (29/30)	3.3% (1/30)
Total	150	98.7% (148/150)	1.3% (2/150)

### Antibiotic resistance profiles

**Figure 3:**
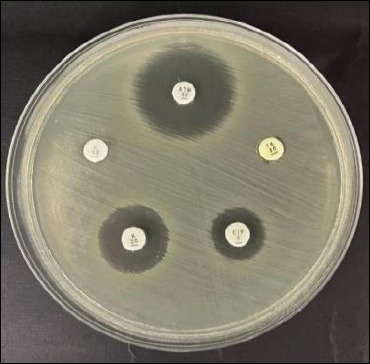
Antibiotic sensitivity test of *Escherichia coli* isolates: (1) aztreonam, (2) tetracycline, (3) ciprofloxacin, (4) kanamycin, and (5) chloramphenicol.

Antibiotic susceptibility testing revealed varying levels of resistance among the 148 *E. coli* isolates. The highest resistance was observed to CIP at 33.1% (49/148), followed by TE at 22.3% (33/148), ATM at 13.5% (20/148), K at 6.1% (9/148), and C at 4.7% (7/148) (Table 2; Figure 3). Market-specific resistance patterns showed notable differences; for instance, Kupang market exhibited the highest CIP resistance at 55.2% (16/29), while Benowo had the highest TE resistance at 31.0% (9/29).

**Table 2:** Identification of antibiotic resistance against *Escherichia coli*.

Traditional market	Number of samples	Number of *E. coli*	ATM R	ATM %	CIP R	CIP %	TE R	TE %	K R	K %	C R	C %
Turi	30	30	5	16.7% (5/30)	8	26.7% (8/30)	7	23.3% (7/30)	1	3.3% (1/30)	1	3.3% (1/30)
Bratang	30	30	3	10% (3/30)	7	23.3% (7/30)	6	20% (6/30)	4	13.3% (4/30)	0	0% (0/30)
Cemara Pabean	30	30	6	20% (6/30)	6	20% (6/30)	3	10% (3/30)	3	10% (3/30)	2	6.7% (2/30)
Kupang	30	29	1	3.5% (1/29)	16	55.2% (16/29)	8	27.6% (8/29)	1	3.5% (1/29)	2	6.8% (2/29)
Benowo	30	29	5	17.2% (5/29)	12	41.4% (12/29)	9	31% (9/29)	0	0% (0/29)	2	6.8% (2/29)
Total	150	148	20	13.5% (20/148)	49	33.1% (49/148)	33	22.3% (33/148)	9	6.1% (9/148)	7	4.7% (7/148)

ATM = Aztreonam; CIP = Ciprofloxacin; TE = Tetracycline; K = Kanamycin; C = Chloramphenicol; R = Resistant.

### MDR isolates

Four isolates (2.7%; 4/148) were classified as MDR, demonstrating resistance to three or more antibiotic classes. These MDR isolates were distributed as follows: one from Turi (3.3%; 1/30), two from Cemara Pabean (6.7%; 2/30), and one from Kupang (3.4%; 1/29), with none detected in Bratang or Benowo (Table 3). The resistance patterns among these MDR isolates included ATM/CIP/TE (ATM/CIP/TE) in two isolates (PTU29 and PKU21), ATM/CIP/K (ATM/CIP/K) in one isolate (PCP25), and ATM/CIP/TE/K/C (ATM/CIP/TE/K/C) in one isolate (PCP26) (Table 4).

**Table 3:** Results of multidrug resistance test on *Escherichia coli*.

Traditional market	Number of *E. coli*	Multidrug-resistant	Percentage (%)
Turi	30	1	3.3% (1/30)
Bratang	30	0	0% (0/30)
Cemara Pabean	30	2	6.6% (2/30)
Kupang	29	1	3.4% (1/29)
Benowo	29	0	0% (0/29)
Total	148	4	2.7% (4/148)

**Table 4:** Resistance patterns of multidrug-resistant *Escherichia coli* strains.

Sample code	ATM	CIP	TE	K	C	Resistance pattern
PTU29	R	R	R	S	S	ATM/CIP/TE
PKU21	R	R	R	S	S	ATM/CIP/TE
PCP25	R	R	S	R	S	ATM/CIP/K
PCP26	R	R	R	R	R	ATM/CIP/TE/K/C

ATM = Aztreonam; CIP = Ciprofloxacin; TE = Tetracycline; K = Kanamycin; C = Chloramphenicol; R = Resistant; S = Sensitive.

### Detection of the *iroN* gene

**Figure 4:**
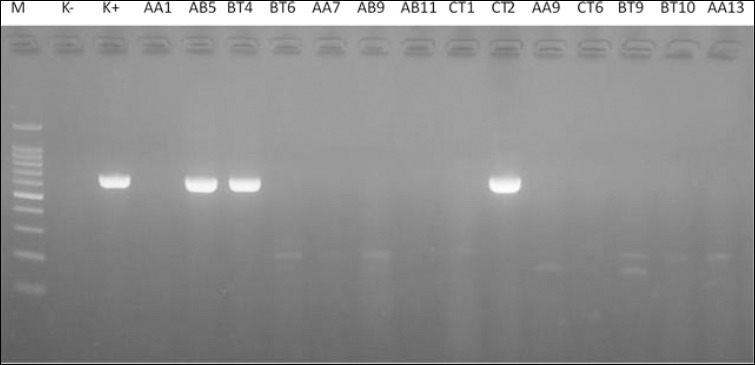
Polymerase chain reaction amplification of iron-related genes in *Escherichia coli* isolates. Clear DNA bands corresponding to the expected amplicon size (667 base pairs) were observed in isolates AB5, BT4, and CT2, indicating positive results. No specific amplification bands were detected in isolates AA1, BT6, AA7, AB9, AB11, CT1, AA9, CT6, BT9, BT10, and AA13, which were considered negative. M: DNA molecular weight marker; K-: negative control; K+: positive control.

**Figure 5:**
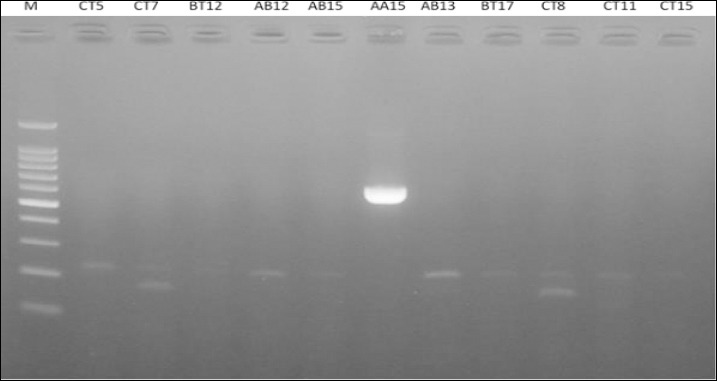
Polymerase chain reaction amplification of iron-related genes in *Escherichia coli* isolates. Clear DNA bands corresponding to the expected amplicon size (667 base pairs) were observed in isolate AA15. No specific amplification bands were detected in isolates CT5, CT7, BT12, AB12, AB15, AB13, BT17, CT8, CT11, and CT15, which were considered negative. M: DNA molecular weight marker; K-: negative control; K+: positive control.

PCR analysis for the *iroN* gene was performed on the four MDR isolates, resulting in amplicons of the expected 861 bp size for all samples, confirming 100% positivity (Figures 4 and 5). This indicates a complete association between MDR phenotypes and the presence of the *iroN* virulence gene in the isolated strains.

## DISCUSSION

### Prevalence and characteristics of *E. coli* in quails

The results of this study indicate a very high prevalence of *E. coli* in quail cloacal swab samples sold in five traditional markets in Surabaya. The high isolation rate of *E. coli* (98.7%) indicates that quail sold in environments with limited sanitation can be important reservoirs for enteric bacteria, including APEC. This finding is consistent with previous studies reporting a high prevalence of Gram-negative bacteria in poultry sold in traditional markets, which is generally caused by cross-contamination, high animal density, and inadequate hygiene facilities [25]. Similar high prevalence rates have also been reported in quail from Spain and Iran, suggesting that quail may serve as consistent reservoirs of *E. coli* globally [26, 27]. Epidemiologically, these conditions increase the risk of transmission of pathogenic bacteria both between animals and from animals to humans. Although this study did not directly assess human infection, the zoonotic risk remains potential rather than confirmed [28].

### Confirmation of *E. coli* identity

Confirmation of *E. coli* identity through growth characteristics on EMBA, Gram staining, and biochemical tests showed a pattern consistent with the classic description of *E. coli*. Lactose fermentation, TSIA results with A/A reaction, positive indole, positive MR, and negative VP are highly characteristic combinations of parameters. This confirms that the isolates obtained are truly representative of *E. coli*, providing a solid basis for further analysis regarding antibiotic resistance and virulent gene detection [29].

### AMR patterns

The antibiotic resistance profile shows varying levels of resistance between markets, but overall, it shows a concerning pattern, particularly against CIP (33.1%), TE (22.2%), and ATM (13.5%). The high level of CIP resistance is significant, given that fluoroquinolones are key first-line antibiotics for treating Gram-negative bacterial infections in humans and animals [30]. The high level of TE resistance also reflects uncontrolled antibiotic use in the poultry sector, as TE is one of the most commonly used antibiotics as a feed additive and for the treatment of infections [31]. Meanwhile, the relatively low levels of resistance to K and C may reflect their limited use in the field [32].

### Differences in resistance levels between markets

Differences in resistance levels between markets indicate variations in drug management practices and sanitation in the marketing environment. For example, the high levels of CIP resistance in Kupang (55.1%) and Benowo (41.3%) may reflect the excessive antibiotic exposure of poultry supplied to these markets. Other possible contributing factors include farm-level poultry husbandry practices, differences in suppliers, and the transport conditions of animals before they reach the market [33].

### Multidrug resistance

Although only 2.7% of the total isolates were classified as MDR, MDR strains remain a serious health threat. The relatively low MDR prevalence despite high single-drug resistance may reflect the limited accumulation of multiple resistance determinants in quail-derived *E. coli* compared with broilers or layers, possibly due to shorter-term antibiotic exposure [34]. MDR isolates were found in three markets, indicating a non-localized distribution [35]. The most common MDR pattern was resistance to a combination of ATM, CIP, and TE. Meanwhile, one isolate (PCP26) showed the most broad resistance to all the tested antibiotics. Although this broad resistance pattern is often associated with large plasmids carrying multiple resistance genes, plasmid analysis was not performed in this study, so the plasmid-mediated mechanism remains speculative [36].

### Antibiotic resistance patterns variation

The fact that all isolates with MDR patterns showed resistance to ATM aligns with research findings showing that resistance to monobactam antibiotics can be an indicator of strong antibiotic selection pressure and the mobilization of genetic factors in the farming environment [37]. Resistance to CIP and TE along with MDR also reflects common patterns of antibiotic use in the poultry sector [38]. Antibiotic resistance patterns varied between markets, suggesting the presence of localized pressures on antimicrobial selection. These differences may arise from distinct supply chains, varying hygiene standards, and differences in the antimicrobial exposure histories of poultry supplied to each market [39]. Such inter-market variation underscores the existence of micro-ecological AMR niches within urban markets, emphasizing the importance of stratified monitoring to identify points of heightened resistance emergence [40].

### Presence of virulence genes

The presence of virulence genes associated with the iron acquisition system provides important insights into the potential pathogenicity of MDR *E. coli* in quail [3]. Four of the 20 ATM-resistant isolates were detected to carry the *iroN* gene in this study. This gene is a crucial component of the iron transport system that enables bacteria to survive iron-limited conditions in the host [16]. This strengthens the hypothesis that antibiotic selection pressure not only drives the emergence of resistance but may also correlate with the high prevalence of virulence factors [41].

### Link between antibiotic resistance and virulence

The presence of the *iroN* gene in some MDR isolates suggests a possible biological link between antibiotic resistance and virulence [42]. However, the mechanistic linkage between iron acquisition and AMR was not directly tested in this study; therefore, it remains hypothetical. Large plasmids found in APEC are often “hybrid,” meaning they carry a combination of resistance and virulence genes, including siderophore systems such as aerobactin and salmochelin [43]. Thus, *E. coli* isolates carrying the *iroN* gene may have stronger colonization and invasion capabilities, increasing their potential to cause systemic infections in poultry [44]. The detection of the *iroN* gene in a subset of MDR *E. coli* isolates highlight the emerging concept of resistance–virulence convergence. Not all resistant isolates carried *iroN*, suggesting that rather than indicating a universal linkage, antibiotic selection may selectively enrich isolates with both resistance and survival/virulence traits [45]. This observation emphasizes the potential of certain MDR subpopulations to harbor adaptive traits that enhance fitness in crowded market environments, providing an evolutionary and ecological perspective beyond simple descriptive microbiology [46].

### Role of the *iroN* gene

In addition to its role in pathogenicity, the *iroN* gene functions as a key determinant of bacterial survival under iron-limited and antibiotic-stressed conditions. Its presence may enhance environmental persistence, stress tolerance, and competitive fitness, particularly in the crowded and unsanitary conditions of traditional markets [45]. Therefore, the detection of *iroN* in MDR *E. coli* not only reflects virulence potential but may also indicate enhanced survival and dissemination capacity, highlighting the importance of iron acquisition systems in shaping bacterial fitness in market level ecological niches. This study suggests that iron acquisition systems contribute to the environmental fitness of MDR *Escherichia coli* in traditional market settings [47].

### Implications for public health and One Health

The results confirm that quail in traditional markets in Surabaya, including MDR strains carrying virulence genes related to iron acquisition, are a significant reservoir for APEC. While this suggests potential public health relevance, direct human transmission was not assessed in this study. This has important implications for animal health, public health, and food safety [48]. The One Health approach is essential for monitoring the flow of resistance and virulence genes from the poultry sector to humans [49]. Strengthening antibiotic management, improving market hygiene, and ongoing molecular monitoring are needed to prevent further spread of the disease [50]. From the perspective of One Health, quail sold in traditional markets may serve as a conduit for MDR and virulence gene dissemination [51]. Potential human exposure pathways include direct handling of live birds, slaughtering practices within the markets, and contact with contaminated surfaces or waste, which can facilitate cross-species transmission [52]. This study provides baseline data essential for modeling One Health risks and designing targeted interventions by characterizing MDR and iron acquisition traits at this market interface.

## CONCLUSION

This study revealed a high prevalence of *E. coli* (98.7%) in cloacal swabs from quails sold in Surabaya’s traditional markets, with isolation rates reaching 100% in three markets (Turi, Bratang, and Cemara Pabean). Antibiotic susceptibility testing showed notable resistance, highest to CIP (33.1%), followed by TE (22.3%), ATM (13.5%), K (6.1%), and C (4.7%), with market-specific variations indicating localized AMR pressures. Four isolates (2.7%) were classified as MDR, exhibiting patterns such as ATM/CIP/TE, ATM/CIP/K, and ATM/CIP/TE/K/C. PCR confirmed that all MDR isolates carried the *iroN* virulence gene, demonstrating a 100% association between MDR phenotypes and this iron acquisition determinant, which enhances bacterial survival and pathogenicity in APEC strains.

These findings underscore quails in traditional markets as potential reservoirs for MDR *E. coli* with zoonotic potential, similar to ExPEC in humans, posing risks to public health through direct handling, cross-contamination, and environmental exposure. Practically, this calls for improved market hygiene, such as enhanced sanitation, waste management, and vendor training, alongside stricter antibiotic stewardship in smallholder poultry farming to curb AMR selection. Within a One Health framework, integrating veterinary surveillance with public health measures could mitigate transmission, informing policies for antimicrobial use regulation and market level interventions in Indonesia.

The study’s strengths lie in its focus on an understudied host (quails) at the market interface, a critical node for pathogen dissemination, using a representative sample from five geographically diverse markets in Surabaya. Employing standardized methods like the Kirby–Bauer assay per CLSI guidelines and PCR for *iroN* detection ensured robust, reproducible results. The cross-sectional design provided a snapshot of real-world AMR and virulence convergence, filling a research gap in Indonesian traditional markets and contributing baseline data for One Health surveillance.

Limitations include the small number of MDR isolates (n = 4), which restricts generalizability, and reliance on phenotypic resistance testing without exploring genotypic mechanisms like plasmid analysis or whole-genome sequencing. The study did not assess direct human transmission or include farm-origin data, potentially overlooking upstream factors. Additionally, sampling was limited to cloacal swabs from apparently healthy quails, excluding tissue or environmental samples that could reveal broader contamination dynamics.

Future research should expand to longitudinal studies tracking AMR evolution, incorporate genomic sequencing to elucidate co-located resistance and virulence genes, and investigate transmission pathways to humans and the environment. Comparative analyses with other poultry types or regions could identify broader patterns, while intervention trials testing hygiene protocols or alternative antimicrobials would evaluate mitigation strategies. Exploring additional virulence genes beyond *iroN* and assessing economic impacts on smallholder farmers would further strengthen One Health approaches.

In conclusion, quails in Surabaya’s traditional markets harbor prevalent *E. coli* with concerning AMR profiles, including MDR strains carrying the *iroN* gene, highlighting their role as reservoirs for potentially zoonotic APEC. These insights emphasize the urgent need for integrated surveillance, hygiene improvements, and prudent antibiotic use to safeguard animal and human health under the One Health paradigm, ultimately reducing the global burden of AMR.

## DATA AVAILABILITY

All data generated or analyzed during this study are included in the manuscript. Additional supplementary data are available from the corresponding author upon reasonable request.

## AUTHORS’ CONTRIBUTIONS

MOK and UR: Conceptualized and supervised the study and drafted the manuscript. JYHT and FJW: Data curation and formal analysis. MHE and ARK: Investigation and visualization. MFRP, BPP, and RZA: Methodology. WW, IAK, and SR: Validation. SR: Review and editing. All authors have read, reviewed, and approved the final manuscript.
